# Shoot and Root Traits Contribute to Drought Resistance in Recombinant Inbred Lines of MD 23–24 × SEA 5 of Common Bean

**DOI:** 10.3389/fpls.2017.00296

**Published:** 2017-03-03

**Authors:** Jose Polania, Idupulapati M. Rao, Cesar Cajiao, Miguel Grajales, Mariela Rivera, Federico Velasquez, Bodo Raatz, Stephen E. Beebe

**Affiliations:** Centro Internacional de Agricultura TropicalCali, Colombia

**Keywords:** deep rooting, intermittent drought, pod harvest index, root system, seed number

## Abstract

Drought is the major abiotic stress factor limiting yield of common bean (*Phaseolus vulgaris* L.) in smallholder systems in Latin America and eastern and southern Africa; where it is a main source of protein in the daily diet. Identification of shoot and root traits associated with drought resistance contributes to improving the process of designing bean genotypes adapted to drought. Field and greenhouse studies were conducted at the International Center for Tropical Agriculture (CIAT), Palmira, Colombia to determine the relationship between grain yield and different shoot and root traits using a recombinant inbred lines (RILs) population (MD23–24 × SEA 5) of common bean. The main objectives of this study were to identify: (i) specific shoot and root morpho-physiological traits that contribute to improved resistance to drought and that could be useful as selection criteria in breeding beans for drought resistance; and (ii) superior genotypes with desirable shoot and root traits that could serve as parents in breeding programs that are aimed at improving drought resistance. A set of 121 bean genotypes (111 RILs, 2 parents, 8 checks) belonging to the Mesoamerican gene pool and one cowpea variety were evaluated under field conditions with two levels of water supply (irrigated and rainfed) over three seasons. To complement field studies, a greenhouse study was conducted using plastic cylinders with soil inserted into PVC pipes, to determine the relationship between grain yield obtained under field conditions with different root traits measured under greenhouse conditions. Resistance to drought stress was positively associated with a deeper and vigorous root system, better shoot growth, and superior mobilization of photosynthates to pod and seed production. The drought resistant lines differed in their root characteristics, some of them with a vigorous and deeper root system while others with a moderate to shallow root system. Among the shoot traits measured, pod harvest index, and seed number per area could serve as useful selection criteria for assessing sink strength and for genetic improvement of drought resistance in common bean.

## Introduction

Drought is the main abiotic constraint of common bean (*Phaseolus vulgaris* L.) affecting around 60% of bean producing regions and causing 10–100% reduction in production (Rao, 2014; Polania et al., 2016c). Beans are cultivated by small farmers in Latin America and eastern and southern Africa, under unfavorable climate conditions and minimum use of inputs (Beebe et al., 2014; Rao, 2014). It is expected that the world demand for legumes will increase in the future, not only in developing countries, but also in the developed nations given the trend toward healthier diets (Daryanto et al., 2015). Common beans have to confront climate change and the associated increase of temperature and evapotranspiration together with erratic and lower rainfall (Beebe et al., 2013; Polania et al., 2016a,b; Rippke et al., 2016). The development of bean varieties resistant to drought stress conditions through breeding to ensure food security in marginal areas is a useful strategy to face these new challenges.

Conventional breeding for improving resistance to drought has been based essentially in the selection of the superior genotypes in grain yield (GY) under drought stress (Rosales et al., 2012); with low consideration for defining the physiological basis of drought resistance. The integration and the understanding of the physiological basis of yield limitations due to drought stress, will contribute to developing physiological selection tools to support plant breeding programs (Araus et al., 2002; Girdthai et al., 2009; Mir et al., 2012). Some of the benefits from improved understanding and use of the physiological traits and mechanisms would be the possibility of combining parents with complementary traits, resulting in additive gene action for improving drought resistance (Reynolds and Trethowan, 2007; Mir et al., 2012).

Phenotypic characterization for drought resistance has resulted in the identification of some morpho-physiological traits and process related to improved drought resistance. Processes that are known to influence drought resistance include: more acquisition of water by the root system from the soil profile to facilitate transpiration, greater production of canopy biomass (CB) and an efficient and increased mobilization of accumulated carbon to the harvestable product (Passioura, 1997; Condon et al., 2004; Polania et al., 2016a; Rao et al., 2016a). Several traits have been reported to improve resistance to drought, and their contribution to superior GY depends on the type of drought (early, intermittent, and terminal) and the agro-ecological conditions where the crop is planted. Ideotypes and plant models have been developed for targeting in plant breeding according to agro-ecological zones and types of drought; for example, the isohydric (“water saving”) plant model and the anisohydric (“water spending”) plant model. The water saving ideotype might have an advantage in the harsh environments, whereas the water spending ideotype will perform relatively better under more moderate drought conditions (Blum, 2015; Polania et al., 2016a).

It has been reported that traits related with higher water use efficiency (WUE) and conserving water at vegetative stage (lower leaf conductance, smaller leaf canopy), would make more water available for reproductive growth and grain filling, resulting in better grain yield under terminal drought stress conditions (Zaman-Allah et al., 2011; Araújo et al., 2015). Increased WUE, could have a penalty in GY, to reduce the rate of transpiration and crop water use, processes that are crucial for carbon assimilation (Blum, 2009; Sinclair, 2012). Blum (2009) proposed the term, effective use of water (EUW), which implies maximal soil moisture capture for transpiration, and also involves decreased non-stomatal transpiration and minimal water loss by soil evaporation. In the water spending model, the EUW would be the main component to consider in plant breeding program for drought resistance, and it is relevant when there is still soil water available at maturity or when deep-rooted genotypes access water deep in the soil profile that is not normally available (Araus et al., 2002; Polania et al., 2016a).

Previous research on common bean under drought stress has suggested the relevance of “water spending” model for improving drought resistance through EUW. Positive relationships have been observed between GY and carbon isotope discrimination (CID) and also with root length density in different genotypes grown under drought stress; indicating that plants under drought stress access more water, resulting in increased stomatal conductance and higher GY (Sponchiado et al., 1989; White et al., 1990; White, 1993; Hall, 2004; Polania et al., 2012, 2016a). Phenotypic evaluation of root traits under drought stress has shown the contribution of deep rooting to access more water from deeper soil layers (Sponchiado et al., 1989; White and Castillo, 1992; Polania et al., 2009, 2012; Beebe et al., 2013, 2014; Lynch, 2013; Rao, 2014; Rao et al., 2016b) and also increased production of fine roots in top soil to take advantage of intermittent rains (Eissenstat, 1992; Huang and Fry, 1998; Polania et al., 2009; Butare et al., 2011; Lynch, 2013; Beebe et al., 2014; Rao et al., 2016a,b).

Increased water extraction capacity and higher crop growth must be accompanied by an improved harvest index (HI) to increase drought resistance. Better remobilization of photosynthates to grain production is needed for the success of superior genotypes under stress. In the case of common bean, the contribution of superior remobilization of reserves from vegetative plant structures to pod and seed formation have been widely documented (Assefa et al., 2013; Beebe et al., 2013; Rao et al., 2013, 2016a; Rao, 2014; Polania et al., 2016a,c). Two dry matter partitioning indices have been shown to be relevant to improved drought resistance: pod partitioning index (PPI) which indicates the extent of mobilization of assimilates from the vegetative structures to pod formation, and pod harvest index (PHI) which indicates the extent of mobilization of assimilates from the pod wall to grain formation (Rao et al., 2013). Photosynthate supply could exert significant and positive quantitative influence on sink strength through setting of grain and pod numbers because abortion rates were shown to be positively related to seed size (Lord and Westoby, 2012).

The strategic combination of specific shoot and root traits seems to be the key in improved resistance to drought in common beans, and no single trait was identified for its unique and dominant contribution to drought resistance (Polania et al., 2016a,c). For that reason, it is relevant to evaluate different shoot and root traits in a same group of genotypes under drought stress as well as under optimal conditions. This will allow to identify the traits that are not only contributing to improved drought resistance but also responding to irrigation. Most of the lines identified with superior drought resistance in common bean are from the Mesoamerican gene pool, where some lines from Durango race were found to be far superior in their drought resistance (Beebe et al., 2013). For example, the drought resistant line SEA 5 is derived from Durango race and it showed greater ability for remobilization of photoassimilates contributing to higher GY under drought conditions (Beebe et al., 2013; Polania et al., 2016c; Rao et al., 2016a).

The main objectives of this study were to identify: (i) specific shoot and root morpho-physiological traits that contribute to improved resistance to drought and that could be useful as selection criteria in breeding beans for drought resistance; and (ii) superior genotypes with desirable shoot and root traits that could serve as parents in breeding programs that are aimed to improve drought resistance.

## Materials and methods

### Plant material

For this study a total of 121 (but 118 for comparison) bean genotypes belonging to the Mesoamerican gene pool and one cowpea variety were selected: 111 RILs of MD 23–24 × SEA 5, two parents (MD 23–24 and SEA 5), and eight checks [Cowpea cv. Mouride, Tio Canela 75, DOR 390, EAP 9510-77, SEA 5 (twice), MD 23–24, and SEA 15]. Cowpea genotype was included for relative comparison of common bean with cowpea for drought resistance. The line MD 23–24 is superior in commercial grain quality and it is also known as “Bribri,” it is small red bean, developed by the Escuela Agricola Panamericana (EAP), Zamorano, Honduras, and released as a good yielding, well adapted to low soil fertility, and disease resistant cultivar (Rosas et al., 2003). The CIAT bred line SEA 5 is very well adapted to drought, it has small (22–25 g 100 seed^−1^) cream-colored seeds and Type III growth habit; also resistant to *Fusarium* root rot and has the I gene for resistance to bean common mosaic virus (BCMV). It is susceptible to anthracnose, common bacterial blight, and rust (Singh et al., 2001). The progenies from the cross were advanced by bulk method up to F4 generation and then to two more generations (F5, F6) by pedigree method followed by bulking in F7 generation.

### Shoot phenotyping under field conditions

#### Experimental site and meteorological conditions

**Figure 1:**
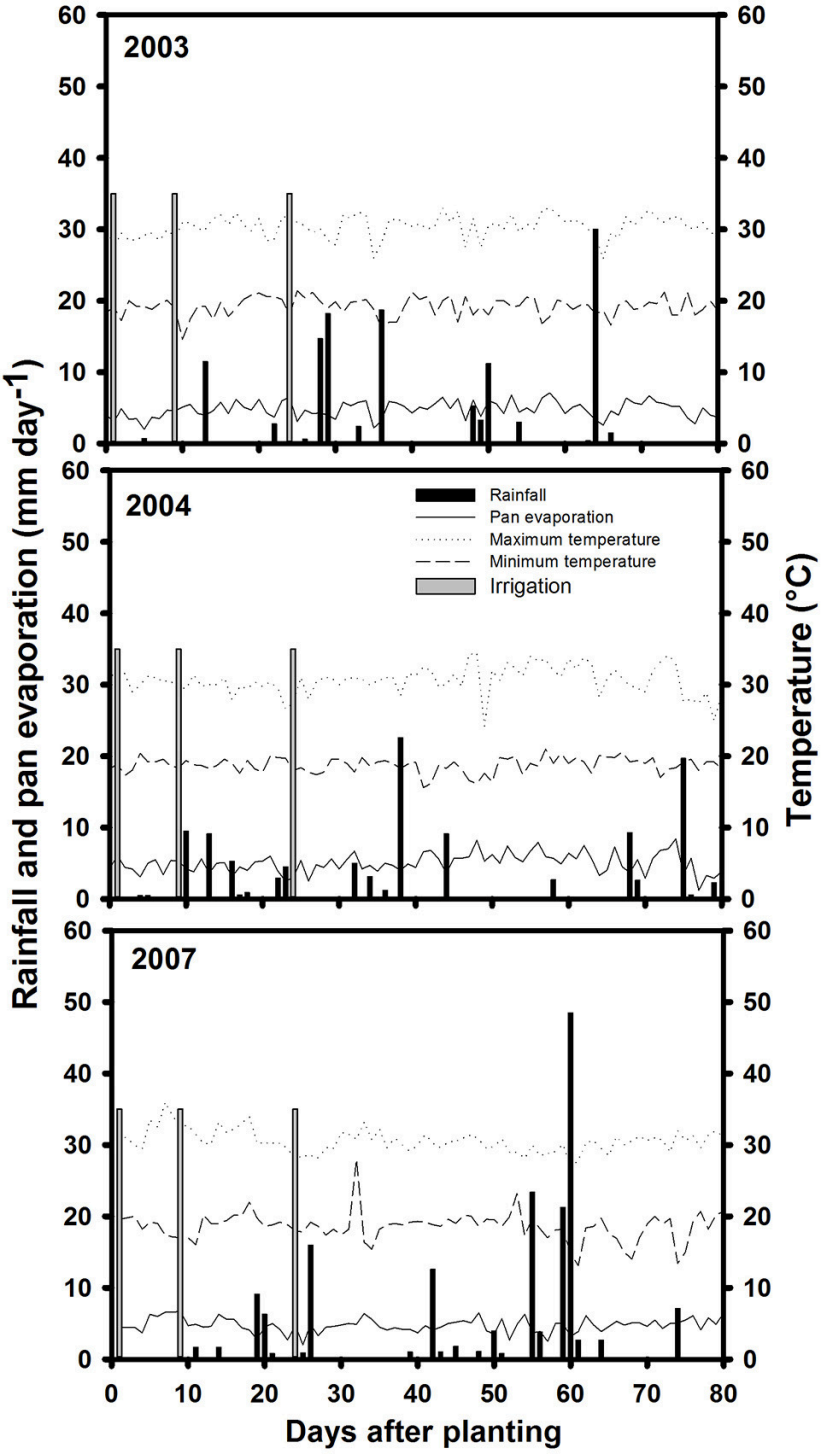
**Rainfall distribution and irrigation application, pan evaporation, maximum, and minimum temperatures during crop growing period at Palmira during 2003, 2004, and 2007 crop growing seasons**.

Three field trials were conducted during the dry season (from June to September in each year of 2003, 2004 and 2007), at the main experiment station of the International Center for Tropical Agriculture (CIAT) in Palmira, Colombia, located at 3° 29″ N latitude, 76° 21″ W longitude and an altitude of 965 masl. Basic characteristics of this field site were described previously (Beebe et al., 2008). The soil is a Mollisol (Aquic Hapludoll) with adequate nutrient supply and is estimated to permit storage of 100 mm of available water (assuming 1.0 m of effective root growth with −0.03 and −1.5 MPa upper and lower limits for soil matric potential). During the crop-growing season in field conditions, maximum and minimum air temperatures in 2003 were 33 and 14.6°C, in 2004 were, 34.4 and 15.6°C and in 2007 were, 30.5 and 18.6°C, respectively (Figure 1). The total rainfall during the active crop growth was 126.5 mm in 2003, 110.4 mm in 2004 and 243.1 mm (a significant proportion of which fell during seed filling) in 2007. The pan evaporation was of 363 mm in 2003, 390 mm in 2004, and 431 mm in 2007. These data on rainfall and pan evaporation together with rainfall distribution indicated that the crop suffered intermittent drought in all 3 years during active growth and development. Two levels of water supply (irrigated and rainfed) were applied to simulate well-watered (control) and drought stress treatments. Trials were furrow irrigated up to 100% field capacity (approximately 35 mm of water per irrigation). The drought stress treatment under rainfed conditions received irrigations at 3 days before planting and at 9 and 23 days after planting. Irrigation was suspended after the third irrigation to induce drought stress conditions. For the non-stress or irrigated (control) treatment, the crop was irrigated until physiological maturity with a total of six irrigations in 2003 and seven irrigations in both 2004 and 2007.

#### Experimental design

We used an 11 × 11 partially balanced lattice design with three replications in all three seasons. Details on planting and management of the trial were similar to those reported before (Beebe et al., 2008). Experimental units consisted of 4 rows, 3.72 m long by 0.6 m wide with 7 cm between plants in the row (equivalent to 24 plants m^−2^). Trials were managed by controlling weeds with application of herbicides (Fomesafen, Fluazifop-p-butil, and Bentazon) and pests and diseases by spraying with insecticides (Thiametoxam, Clorpirifos, Imidacloprid, Abamectina, Cyromazine, and Milbemectin) and fungicides (Benomil and Carboxin) as needed.

#### Yield measurements and phenological assessment

Grain was harvested from two central rows after discarding end plants in both the irrigated and drought plots. In order to compare shoot dry biomass with grain dry weight and to quantify dry matter distribution among plant parts, mean values of grain yield per hectare were corrected for 0% moisture in grain. Days to physiological maturity (DPM) were determined for each plot as the number of days after planting until 50% of plants have at least one pod losing its green pigmentation.

#### Physiological measurements under field conditions

At mid-pod filling, a 50 cm segment of the row (equivalent to an area of 0.3 m^2^) from each plot with about 7 plants was used for destructive sampling to measure leaf area index (LAI), canopy biomass (CB) and dry matter distribution between leaves, stems and pods. Leaf area was measured using a leaf area meter (model LI-3000, LI-COR, NE, USA) and the leaf area index (LAI) was calculated. Also, at mid-pod filling SPAD chlorophyll meter readings (SCMR) were made on a fully expanded young leaf of three different plants within each replication by using a non-destructive, hand-held chlorophyll meter (SPAD-502 Chlorophyll Meter). At the time of harvest, plants in 50 cm of a row from each plot were cut and dry weights of stem, pod, seed, pod wall, seed number per area (SNA) per m^2^, and pod number per area (PNA) per m^2^ were recorded. The following attributes were determined according to Beebe et al. (2013): harvest index (HI) (%): seed biomass dry weight at harvest/total shoot biomass dry weight at mid-pod filling × 100; pod harvest index (PHI) (%): seed biomass dry weight at harvest/pod and seed biomass dry weight at harvest × 100; PPI (%): pod and seed biomass dry weight at harvest/total shoot biomass dry weight at mid-pod filling × 100. HI and PPI were estimated using the CB-value at mid-pod filling growth stage which is assumed to be the time that reflects the maximum vigor of the genotype; from this time common bean begins to lose CB through leaf fall, especially under drought stress.

### Root phenotyping using soil cylinder system

#### Experimental conditions

A greenhouse study was conducted at CIAT using an Andisol from the region of Darien, Colombia, and mixed (2:1 w/w) with river sand. Soil cylinders were carefully packed with soil:sand mixture, with a final bulk density of 1.2 g cm^−3^. Soil was fertilized with adequate levels of nutrients (kg/ha: 40 N, 50 P, 100 K, 101 Ca, 29 Mg, 20 S, 2 Zn, 2 Cu, 0.1 B, and 0.1 Mo) at planting by mixing with the soil. The seeds were germinated and uniform seedlings were selected for transplanting to transparent plastic cylinders (80 cm long, 7.5 cm diameter), each of which was inserted into PVC sleeve-tubes (Polania et al., 2009). Plants were grown for 48 days in these soil cylinders with an average maximum and minimum temperature of 34° and 21°C.

#### Experimental design

A randomized complete block design (RCB) with three replications was used. Two water supply treatments were applied: (1) well-watered (WW) at 80% field capacity and (2) progressive water stress (WS) with no watering after 10 days of growth in order to simulate terminal drought stress conditions. The initial soil moisture for all the treatments was at 80% of field capacity. The plants with well-watered treatment were maintained close to 80% field capacity by weighing each cylinder every 2 days and applying water to the soil at the top of the cylinder. Plants with progressive soil drying treatment received no water application and each cylinder was weighed at 2 day intervals for the determination of decrease in soil moisture content until the time of plant harvest.

#### Physiological measurements under greenhouse conditions

Plants were harvested at 48 days after transplant (38 days of withholding of water application in the case of drought treatment). Visual rooting depth was measured during the experiment at 7 day intervals using a ruler with cm scale, registering the total length reached by the visible roots of the plastic cylinder. At harvest, leaf area (LICOR model LI-3000), shoot biomass distribution and root distribution were measured. For root distribution traits, the cylinder was sliced into six layers (0–5, 5–10, 10–20, 20–40, 40–60, and 60–75 cm) and the roots in each soil layer were washed free of soil and sand. The washed roots were scanned as images by a desk scanner. From the scanned images, total root length (m plant^−1^), and fine roots proportion (%), were measured using image analysis by WinRHIZO software (Regent Instruments Inc., Quebec, Canada). Root and shoot dry weight was determined after the root and shoot samples were dried in an oven at 60°C for 48 h.

### Statistical analysis

Statistical analysis was performed using the Mixed Procedure of SAS V9.2 (SAS Institute Inc, 2008) and Sigma plot. For the purposes of estimating adjusted line means and comparing check entries with experimental lines, entries, environments, and their interactions were considered fixed effects. Replications and blocks within replications were considered random effects. The mixed model uses information on means of fixed effects that are contained in the differences between blocks, combining the traditional information within the block (Proc GLM) with the new information between blocks (Proc Mixed). Significance of differences among genotypes was tested by the Tukey-Kramer method. Although the mixed model does not present an overall standard error, given that the standard errors associated with each genotype were very similar for each variable, an average standard error was calculated to estimate a significant difference for each variable in the two treatments, for the purpose of visualizing comparisons. The relationships between selected parameters were investigated using the Pearson's correlation test (level of probability at 0.05, 0.01, and 0.001). The principal component analysis (PCA) was used to determine the relationship between multiple variables using PRINCOMP of SAS V9.2 (SAS Institute Inc, 2008). PCA permits creating values that reflect the combined effect of multiple variables that are acting in a similar way.

## Results

### Phenotypic evaluation of shoot traits under field conditions

**Figure 2:**
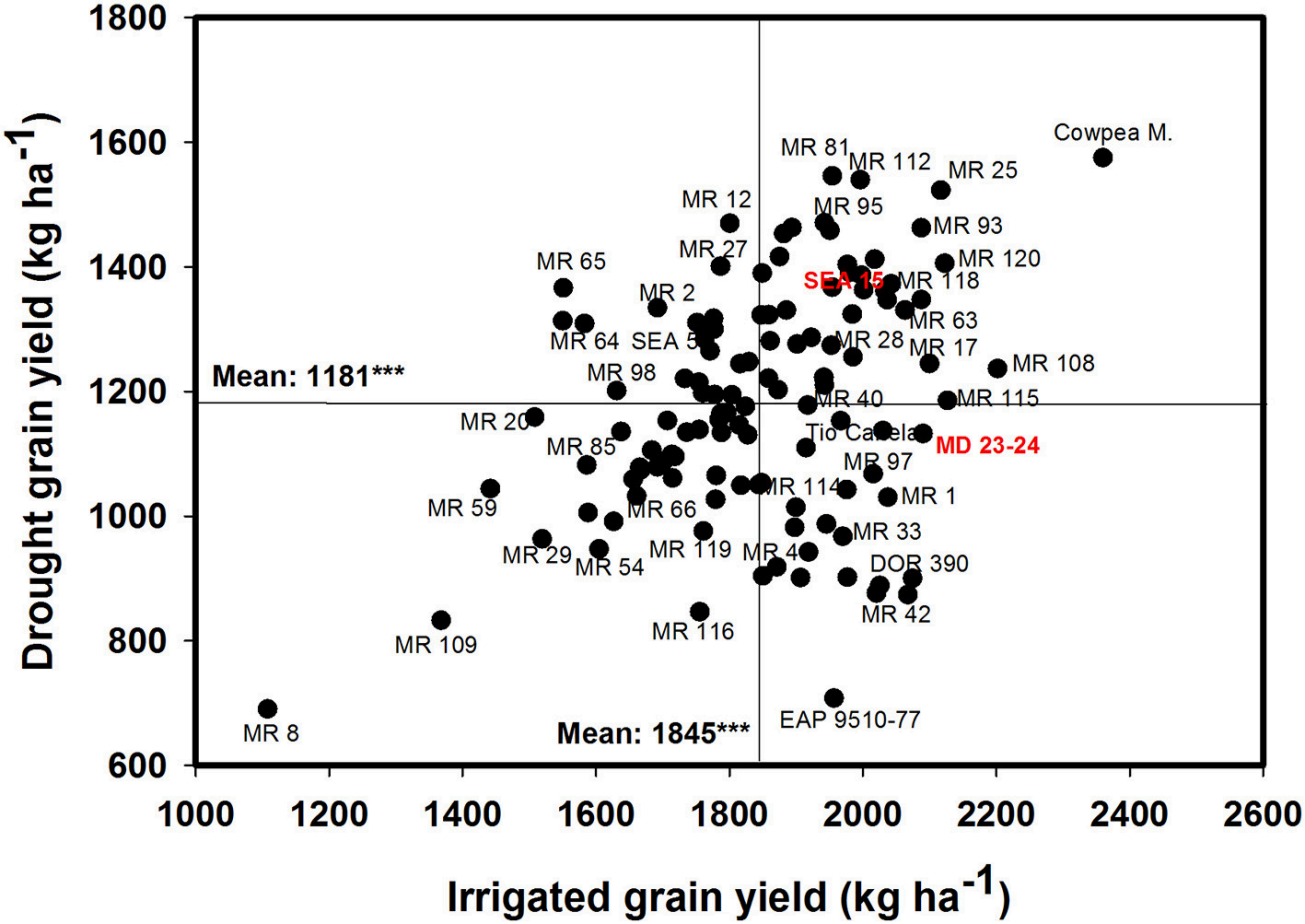
**Identification of genotypes that are adapted to drought stress conditions and are responsive to irrigation in a Mollisol at Palmira**. Genotypes that yielded superior with drought and were also responsive to irrigation were identified in the upper, right hand quadrant.

The data on rainfall distribution, irrigation application, and pan evaporation in both trials indicated that the crop suffered intermittent drought stress during crop development under rainfed conditions (Figure 1). The mean value of GY under drought stress conditions was 1,181 kg ha^−1^ compared with the mean irrigated GY of 1,845 kg ha^−1^ with about 36% reduction of mean grain yield under drought stress (Figure 2). Under drought stress conditions in the field, the GY of 118 genotypes ranged from 690 to 1,575 kg ha^−1^ (Figure 2). Among the lines tested, three RILs, MR 81, MR 112, and MR 25 were outstanding in their adaptation to drought stress conditions. These three lines were also responsive to irrigation. The relationship between GY under drought and irrigated treatments indicated that several RILs were superior to the best parent, SEA 5, and the four common bean check genotypes. Among the 118 genotypes tested, MR 8 was the most poorly adapted RIL under drought stress conditions.

**Figure 3:**
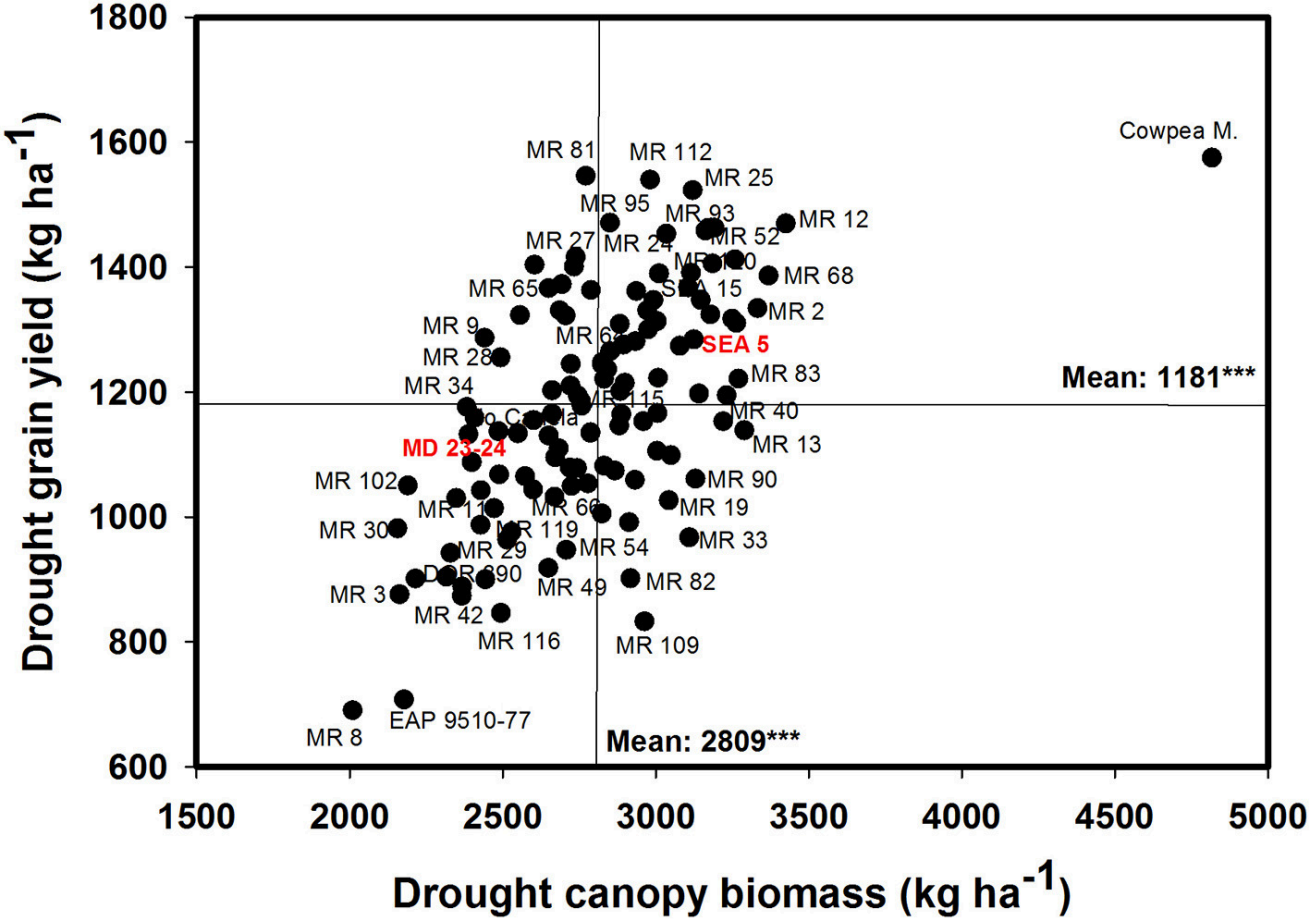
**Relationship between grain yield (GY) and canopy biomass (CB) under drought stress when grown in a Mollisol at Palmira**. Genotypes with higher values of GY and CB under drought conditions were identified in the upper, right hand quadrant.

A positive and significant correlation was observed between CB and GY under both irrigated and drought conditions with values of 0.52^***^ and 0.60^***^, respectively (Table 1). The genotype Cowpea cv. Mouride showed the highest value of CB under stress conditions (Figure 3). But this genotype showed a lower value of HI. Five lines (MR 112, MR 25, MR 93, MR 12, and MR 52) combined higher CB-values with higher GY-values under drought stress conditions (Figure 3). The line MR81 was outstanding in its grain yield under drought conditions, but its CB-value was not high under stress conditions. The susceptible check DOR 390 and five RILS (MR 8, EAP9510-77, MR 3, MR 42, and MR 116) showed poor adaptation to drought conditions with lower values of CB and GY under drought conditions (Figure 3). The drought adapted parent SEA 5 showed higher values of CB and GY under drought stress conditions than the susceptible parent MD 23–24 (Figure 3).

**Table 1:** **Correlation coefficients (r) between final grain yield (GY) and other plant attributes and canopy biomass (CB) and other plant attributes of RILs of common bean grown under irrigated and drought stress conditions in a Mollisol in Palmira, Colombia**.

**Plant traits**	**Canopy biomass (kg ha**^**−1**^**)**		**Grain yield (kg ha**^**−1**^**)**	
	**Irrigated**	**Drought**	**Irrigated**	**Drought**
SPAD chlorophyll meter readings (SCMR)	0.40^***^	0.03	0.35^***^	0.06
Leaf area index (LAI; m^2^ m^−2^)	0.68^***^	0.56^***^	0.28^**^	0.30^**^
Canopy biomass (CB; kg ha^−1^)	1.00	1.00	0.52^***^	0.60^***^
Pod harvest index (PHI; %)	0.06	0.20^*^	0.44^***^	0.49^***^
Pod partitioning index (PPI; %)	−0.11	0.18^*^	0.15	0.09
Harvest index (HI; %)	−0.38^***^	−0.34^***^	0.05	0.13
100 seed weight (SW; g)	−0.13	0.14	−0.04	0.22^*^
Grain yield (GY; kg ha^−1^)	0.52^***^	0.60^***^	1.00	1.00
Shoot TNC (mg g^−1^)	0.07	0.22^*^	0.32^***^	0.20^*^
Seed TNC (mg g^−1^)	−0.09	0.19^*^	0.08	0.20^*^
Pod number per area (PNA)	0.45^***^	0.25^**^	0.29^**^	0.03
Seed number per area (SNA)	0.64^***^	0.38^***^	0.40^***^	0.34^***^
Days to maturity (DM)	0.22^*^	−0.20^*^	0.09	−0.31^***^
Visual rooting depth (VRD) at flowering (cm plant^−1^)	0.02	0.18	−0.02	0.18
Root length at soil depth 0–5 cm (RL0–5; m plant^−1^)	−0.07	−0.34^***^	−0.11	−0.22^*^
Root length at soil depth 5–10 cm (RL5–10; m plant^−1^)	−0.05	−0.03	−0.22^*^	0.05
Root length at soil depth 10–20 cm (RL10–20; m plant^−1^)	0.03	0.11	−0.08	0.17
Root length at soil depth 20–40 cm (RL20–40; m plant^−1^)	0.09	0.11	−0.01	0.21^*^
Root length at soil depth 40–60 cm (RL40–60; m plant^−1^)	0.11	0.04	−0.02	0.12
Root length at soil depth 60–75 cm (RL60–75; m plant^−1^)	0.16	0.15	0.01	0.11
Total root length (TRL; m plant^−1^)	0.09	0.00	−0.07	0.11
Fine root proportion at soil depth 0–5 cm (FRP0-5; %)	−0.18^*^	−0.47^***^	−0.18	−0.27^**^
Fine root proportion at soil depth 5–10 cm (FRP5–10; %)	−0.21^*^	−0.35^***^	−0.14	−0.11
Fine root proportion at soil depth 10–20 cm (FRP10–20; %)	−0.18	−0.27^**^	−0.06	−0.08
Fine root proportion at soil depth 20–40 cm (FRP20–40; %)	−0.14	−0.22^*^	−0.04	−0.07
Fine root proportion at soil depth 40–60 cm (FRP40–60; %)	0.03	−0.06	0.22^*^	0.08
Fine root proportion at soil depth 60–75 cm (FRP60–75; %)	0.05	0.13	0.03	0.11
Total fine root proportion (TFRP; %)	0.00	−0.04	0.08	0.07

*, **, *** *Significant at the 0.05, 0.01, and 0.001 probability levels, respectively*.

**Figure 4:**
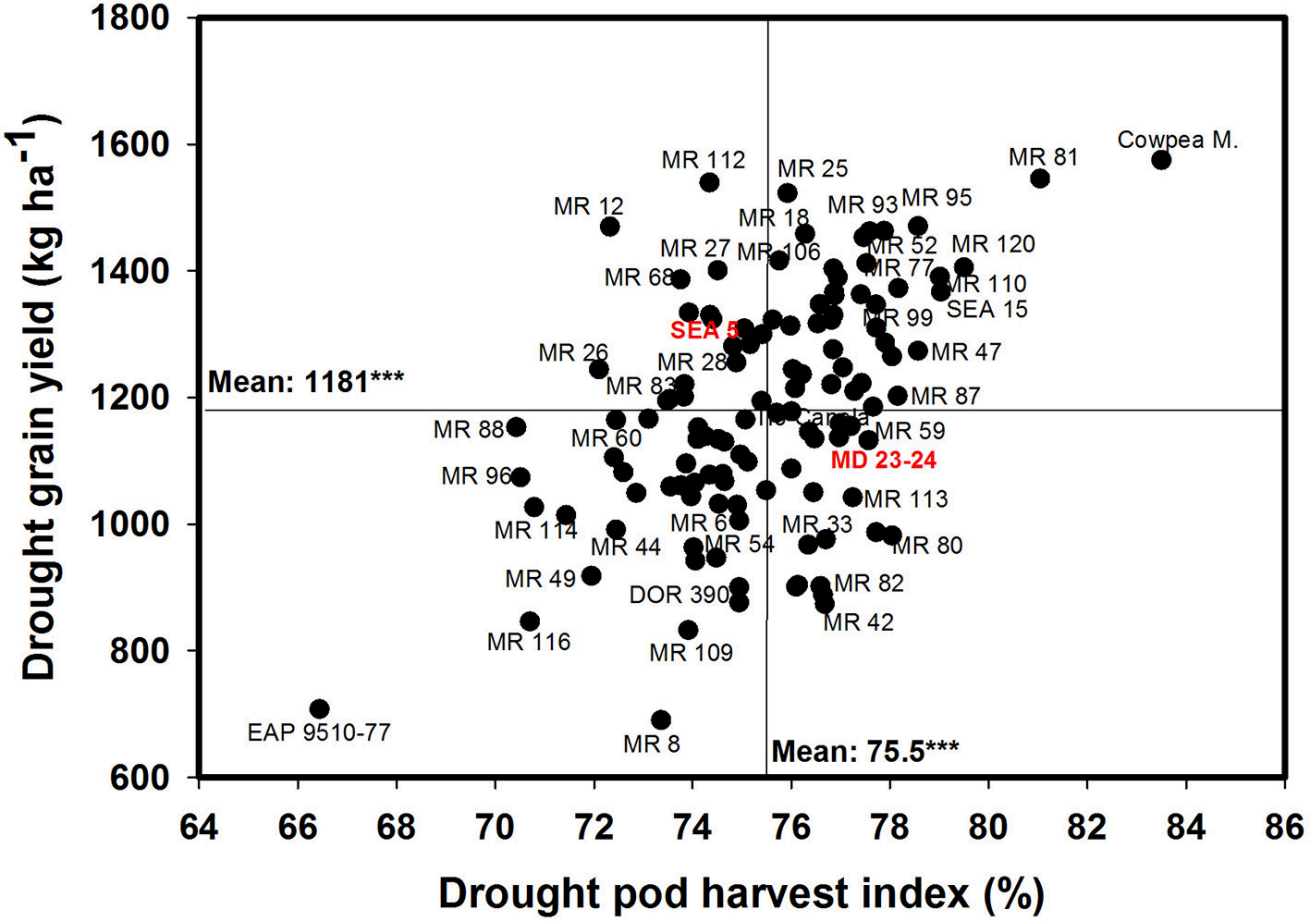
**Relationship between grain yield (GY) and pod harvest index (PHI) under drought stress when grown in a Mollisol at Palmira**. Genotypes with higher values of GY and PHI under drought conditions were identified in the upper, right hand quadrant.

Poor correlation was observed between GY and PPI under drought conditions (Table 1). However, four RILs (MR 12, MR 77, MR 27, and MR 2) combined higher value of PPI and GY under drought stress conditions. Three RILs (MR 34, MR 17, and MR 119) were outstanding in mobilizing photosynthates to pod formation, but the CB-values of these lines were lower under drought stress, which resulted in lower values of GY. The PHI reflects the ability to mobilize photosynthates from pod wall to seed. A positive and highly significant correlation between PHI and GY under both irrigated and drought conditions was observed (Table 1). Nine genotypes (Cowpea, MR 81, MR 95, MR 120, MR 110, SEA 15, MR 52, MR 93, and MR 25) were superior in their ability to mobilize photosynthates from pod wall to grain, resulting in a higher grain yield under drought conditions (Figure 4). Two RILs (MR 112 and MR 12) showed poor performance in photosynthate mobilization to grain formation. Five genotypes (EAP 9510-77, MR 8, MR 116, MR 109, and DOR 390) combined low values of PHI with low values of GY under drought stress (Figure 4). The drought adapted parent SEA 5, with better value of GY than the susceptible parent MD 23–24, presented lower values of PHI than MD 23–24 under drought stress conditions, and slightly below the average of the RILs evaluated. Results on the relationship between the values of GY and HI under drought stress indicated that MR 25 and MR 81 were superior in mobilizing photosynthates to seeds. The HI-values of EAP 9510-77, MR 109 and MR 40 were markedly lower than that of other bean genotypes.

**Figure 5:**
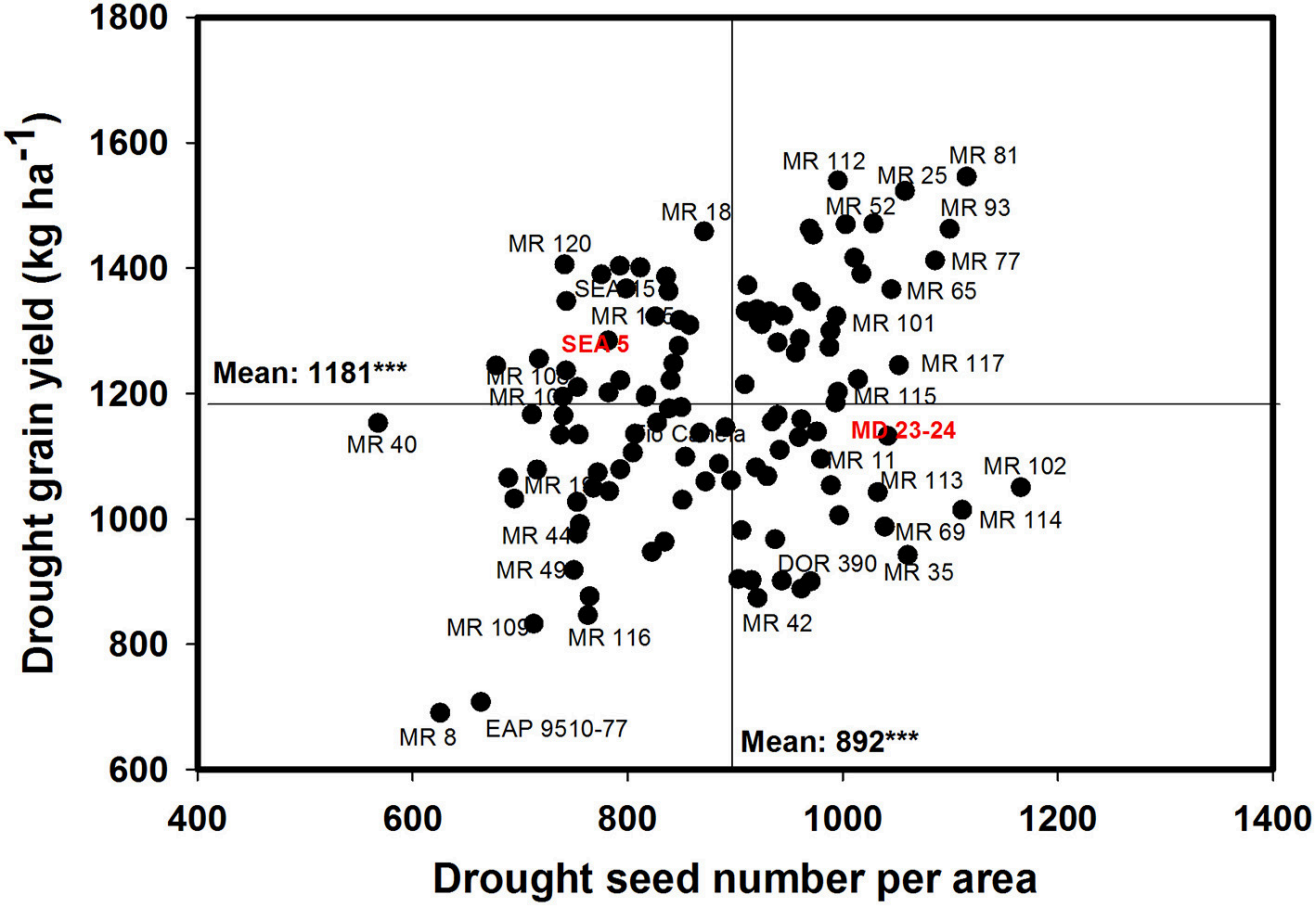
**Relationship between grain yield (GY) and seed number per area (SNA) under drought stress when grown in a Mollisol at Palmira**. Genotypes with higher values of GY and SNA under drought conditions were identified in the upper, right hand quadrant (Cowpea cv. Mouride was excluded in the figure due to its very high seed number value).

A negative and significant correlation (−0.31^***^) was observed between DPM and GY under drought conditions (Table 1). Under irrigated conditions the DPM of 118 genotypes ranged from 62 to 70 days with a mean of 66 days; under drought stress the DPM ranged from 61 to 69 with a mean of 66 days (Data not shown). A total of 22 lines showed shorter and similar DPM under both irrigated and drought conditions (Data not shown). Another group of 22 lines showed shorter DPM with superior values of GY than the other genotypes under drought stress conditions (Data not shown). A negative and significant correlation (*r* = −0.19^*^) was observed between DPM and canopy biomass under drought stress conditions. A positive and significant correlation was observed between DPM and SNA under irrigated and drought conditions (*r* = 0.50^***^ and *r* = 0.31^***^), respectively. The SNA showed a positive and highly significant correlation with grain yield under both irrigated and drought treatments (Table 1). Ten genotypes (Cowpea cv. Mouride, MR 102, MR 81, MR 114, MR 93, MR 77, MR 35, MR 25, MR 117, and MR 65) showed higher values of SNA than the other genotypes under drought stress conditions (Figure 5). Six genotypes (MR 66, MR 92, MR 26, EAP 9510-77, MR 8, and MR 40) were characterized by low values of SNA under drought stress. The parent MD 23–24 presented higher SNA under drought conditions than the parent SEA 5 (Figure 5), but SEA 5 was superior in its 100 seed weight (SW).

**Figure 6:**
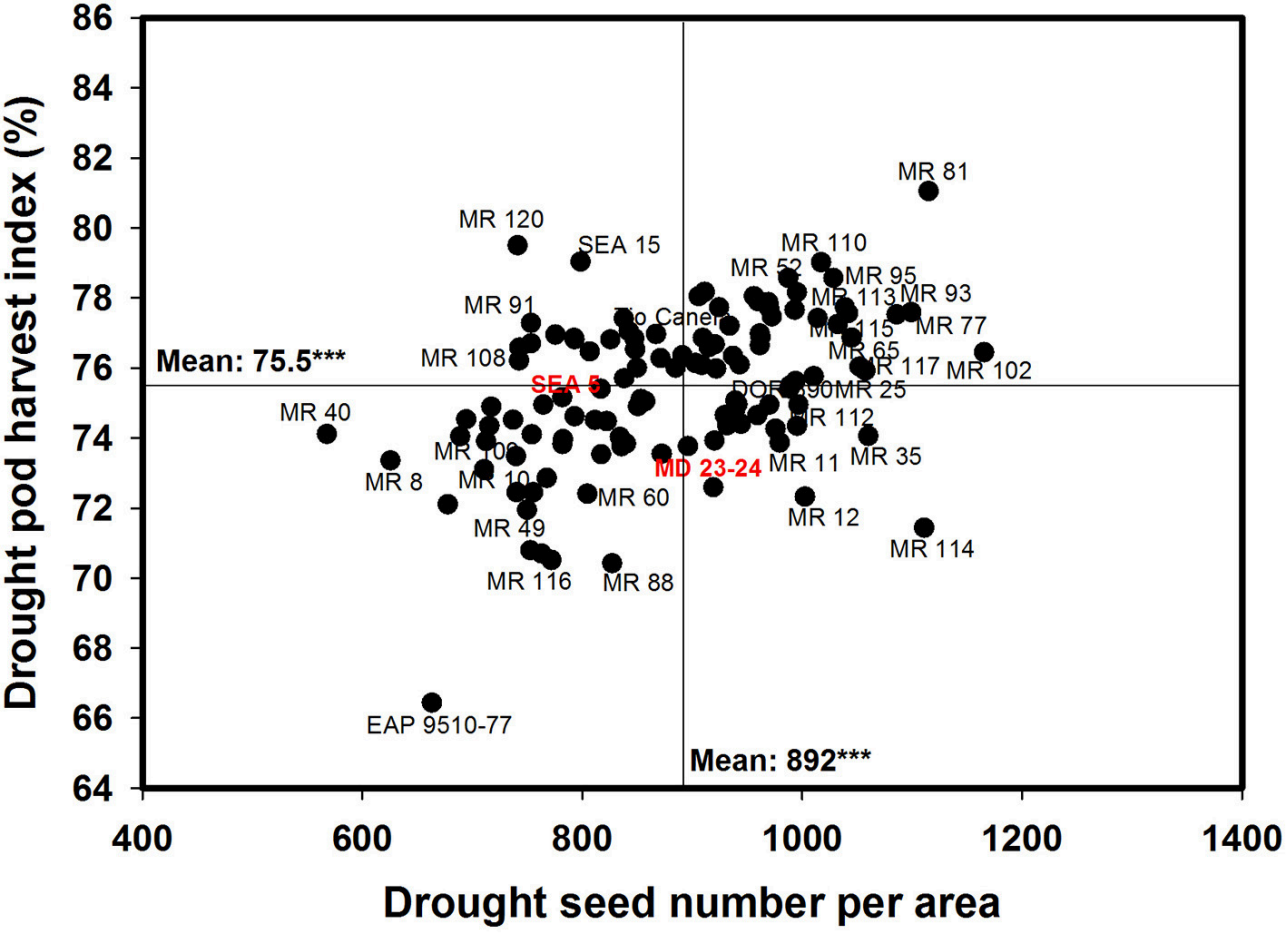
**Relationship between seed number per area (SNA) and pod harvest index (PHI) under drought stress when grown in a Mollisol at Palmira**. Genotypes with higher values of SNA and PHI under drought conditions were identified in the upper, right hand quadrant (Cowpea cv. Mouride was excluded in the figure due to its very high seed number value).

A positive and significant correlation (*r* = 0.52^***^) was observed between SNA and PHI under drought conditions. Five lines (MR 81, MR 110, MR 95, MR 93, MR 120) combined higher SNA with higher PHI values under drought stress conditions (Figure 6). These lines also presented higher grain yield under drought stress (Figure 2). The susceptible check DOR 390, the parent MD 23–24 and five RILS (MR 114, MR 12, MR 11, MR 35) showed higher SNA combined with lower PHI and GY under drought conditions (Figures 2, 6). The lines EAP 9510-77, MR 116, MR 88, MR 49, and MR 8 showed poor adaptation to drought conditions with lower values of SNA, PHI, and GY under drought conditions (Figures 2, 6).

### Phenotypic evaluation of root traits in soil cylinder system

Drought stress reduced root growth in terms of total root length (TRL) compared to irrigated conditions, from an average of 45.9 m plant^−1^ under irrigated conditions to 39.4 m plant^−1^ under drought stress. However, it is noteworthy that several RILs (MR22, MR67, MR87, MR76, MR36, MR104, MR42, MR86, MR107, MR45, MR83, MR85, MR1, MR7, MR80, MR113, MR40, MR66, MR17, MR23, MR33, and MR58) and parents (SEA 5 and MD23–24) were characterized by increased root production under drought stress compared with irrigated conditions (Data no shown). Also an increase in production of deep roots (root length at soil depth 60–75 cm, m plant^−1^) under drought stress was observed for the above mentioned lines (Table 2). Relationship between deep rooting evaluated under greenhouse conditions and GY in field conditions was tested and it didn't show a significant correlation under both irrigated and drought conditions (Table 1).

**Table 2:** **Phenotypic variation in root traits of parents and recombinant inbred lines (RILs) of MD23–24 × SEA 5 grown in soil cylinders under irrigated (well-watered) and drought stress conditions in the greenhouse at Palmira, Colombia**.

**Trait**	**Irrigated**					**Drought**				
	**Parents**		**RILs**			**Parents**		**RILs**		
	**MD 23–24**	**SEA 5**	**Max**	**Min**	**Mean**	**MD 23–24**	**SEA 5**	**Max**	**Min**	**Mean**
Visual rooting depth at flowering (cm plant^−1^)	75	75	75	47	65	73	73	75	44	65
Root length at soil depth 60–75 cm (m plant^−1^)	2.1	2.6	9.0	0.0	3.1	2.7	5.2	6.0	0.0	2.2
Total root length (m plant^−1^)	34	37	78	23	46	43	43	58	21	40
Total fine root proportion (%)	87	84	89	68	81	88	86	90	68	83

**Figure 7:**
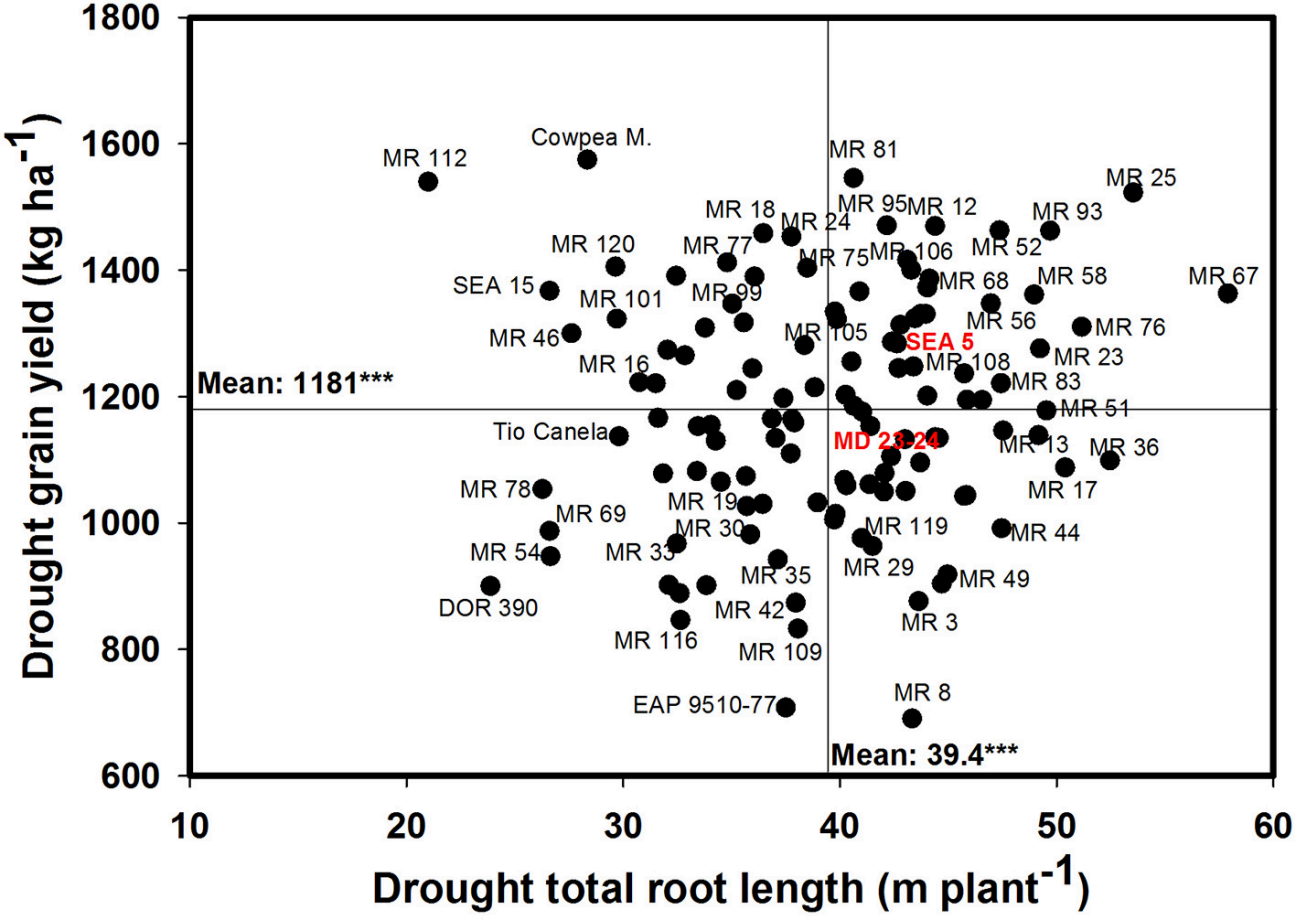
**Relationship between drought grain yield (GY) under field conditions and total root length (TRL) production under drought stress in greenhouse conditions**. Genotypes with higher values of GY and TRL under drought stress were identified in the upper, right hand quadrant.

**Figure 8:**
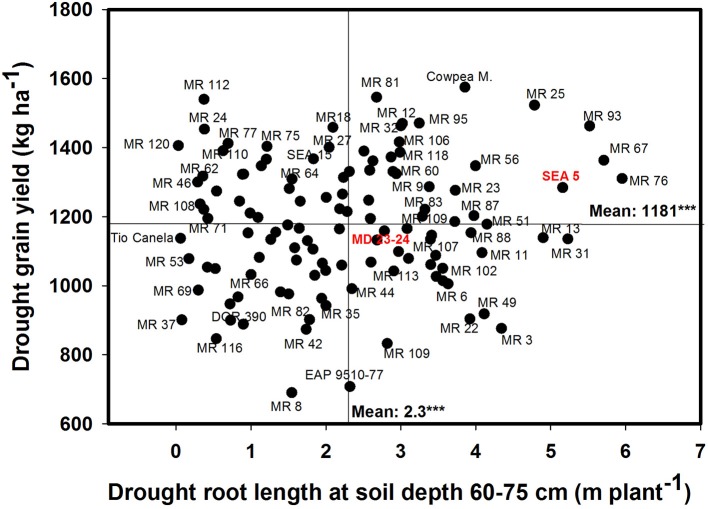
**Relationship between drought grain yield (GY) under field conditions and deep rooting ability (RL60-75) under drought stress in greenhouse conditions**. Genotypes with higher values of GY and RL60–75 under drought stress were identified in the upper, right hand quadrant.

A significant negative correlation (*r* = −0.22^*^) was observed between roots produced at soil depth 0–5 cm with GY under drought stress conditions (Table 1). Also a positive and significant correlation (*r* = 0.21^*^) was observed between roots length production at soil depth 20–40 cm with GY under drought stress (Table 1). A wide range of diversity in TRL was observed under drought conditions (Figure 7). Some genotypes such as one of the parents (SEA 5) and six RILS (MR 81, MR 12, MR 93, MR 25, MR 52, and MR 67) combined vigorous root system with superior values of GY under drought stress, while the three drought sensitive checks (Tio Canela, DOR 390, EAP 9510-77) and five RILs (MR 116, MR 54, MR 78, MR 109, and MR 69) showed poor root growth with lower values of GY under drought stress (Figure 7). Two genotypes (Cowpea, SEA 15) and two RILs (MR 112 and MR 120) were outstanding in their GY under drought stress but had poorer root growth compared with the other lines tested. Contrary to this observation, one parent (MD 23–24) and four RILs (MR 8, MR 3, MR 49, and MR 29) had vigorous root growth but lower GY under drought stress (Figure 7). A strong and positive relationship (*r* = 0.68^***^) between vigorous root growth in term of greater value of TRL, and deep rooting ability in terms of root production at soil depth of 60–75 cm was observed under drought stress. The drought resistant parent SEA 5 was outstanding in its deep rooting ability under drought stress (Figure 8). Several RILs combined deep rooting ability with higher GY under drought stress such as MR 25, MR 93, MR 67, MR 81, MR 95, MR 12, and MR 32 (Figure 8). Three RILs (MR 112, MR 24 and MR 120) showed greater GY and shallow root development under drought stress. Five lines (MR 13, MR 31, MR 49, MR 3, and MR 22) were identified as outstanding in their deep rooting ability under drought stress, but not in producing GY (Figure 8).

A slight increase in the production of fine roots was observed under drought stress compared to irrigation, from an average in fine root proportion of 81% under irrigation to 83% under drought (Table 2). Several RILs were superior to the parents (SEA 5 and MD 23–24) in the increase of fine root production under drought stress. Both parents showed almost similar fine root proportion under both irrigated and drought conditions and were superior to the average value of the RIL population under both conditions (Table 2). No clear relationship between fine root development and grain yield was observed under both irrigated and drought conditions; but under drought stress a significant negative correlation was observed between fine root proportion at different soil layers (0–5, 5–10, 10–20, and 20–40) with CB.

Nineteen RILs (MR11, MR13, MR15, MR19, MR25, MR51, MR6, MR67, MR68, MR79, MR9, MR93, MR98, MR90, MR106, MR3, MR97, MR40, and MR52), and the two parents (SEA 5 and MD 23–24) showed rapid root growth under drought stress, as reflected by the maximum visual rooting depth values under drought conditions (Data not shown). The lower yielding genotypes under drought stress such as DOR 390 and Tio Canela 75 (drought sensitive and commercial checks) were characterized by slow root growth under drought conditions based on the lower values of visual rooting depth. Also these two checks presented a poor root development under drought and well-watered conditions in terms of TRL based on lower values of deep root production (root length at soil depth 60–75 cm, m plant^−1^) and thicker roots under drought stress (Table 2; Figures 7, 8).

### Principal component analysis

Under irrigated and drought stress conditions, eight principal components with cumulative variance of 75% was extracted which gives the clear idea of structure underlying the variables analyzed. Under irrigated conditions for Component 1 which has the contribution of visual rooting depth at flowering, root length at soil depth of 40–60 and 60–75 cm, TRL and fine root proportion for 21% of the total variability. For component 2, SCMR, LAI, CB, SW, GY, PNA, SNA, root length at soil depth of 0–5 cm, root length at soil depth of 10–20 cm, and TRL has contributed to 16% of total variability (Table 3). The principal component analysis (PCA) showed that under irrigated conditions, GY was associated primarily with shorter maturity, PHI, LAI, CB, PNA, SNA, and deep rooting ability. Under drought conditions GY was associated with shorter maturity, PHI, CB, PNA, SNA, deep rooting ability, and thicker roots. Under drought stress conditions for Component 1 which has the contribution of about 22% of the total variability from root traits such as, visual rooting depth at flowering, root length at soil depth of 10–20, 20–40, 40–60, and 60–75 cm, TRL, less fine roots at shallow soil layers and higher fine root proportion at deeper soil layers. For component 2, LAI, CB, SW, PHI, GY, PNA, SNA, DPM, root length at soil depth of 0–5 and 5–10 cm, and TRL have contributed to 14% of total variability (Table 4). PCA also showed that while deep rooting and earliness has contributed to superior performance under drought conditions, the formation of pods and seeds were not the factor limiting the grain yield. It was rather the ability to fill seeds as reflected by the significant positive associations between GY and PHI.

**Table 3:** **Eigen values and percent of total variation and component matrix for the principal component axes—Irrigated conditions**.

**Principal components**	**1**	**2**	**3**	**4**	**5**	**6**	**7**	**8**
Eigen values	5.87	4.49	2.70	2.28	1.85	1.46	1.24	1.15
Proportion of variance	0.21	0.16	0.10	0.08	0.07	0.05	0.04	0.04
Cumulative	0.21	0.37	0.47	0.55	0.61	0.67	0.71	0.75
**COMPONENT MATRIX**								
SCMR	0.094	−0.239	0.096	0.156	−0.041	−0.288	0.020	−0.037
LAI	0.177	−0.247	0.243	−0.062	0.116	−0.163	−0.087	0.247
CB	0.135	−0.265	0.167	0.083	0.388	−0.140	0.086	0.107
PHI	−0.021	−0.118	−0.096	0.373	−0.015	0.316	0.153	−0.053
PPI	−0.078	0.048	−0.056	0.126	0.065	0.289	−0.467	0.456
HI	−0.014	0.000	−0.108	0.192	−0.486	0.209	−0.066	0.146
SW	−0.032	0.288	−0.182	−0.002	0.309	−0.155	0.014	0.263
GY	0.051	−0.214	−0.023	0.385	0.281	0.071	−0.039	0.210
Shoot TNC	−0.107	0.001	−0.151	0.282	0.405	0.051	0.114	−0.267
Seed TNC	−0.061	−0.023	−0.141	0.193	−0.136	0.363	0.394	0.118
PNA	0.090	−0.339	0.192	0.065	−0.139	0.100	0.101	−0.053
SNA	0.141	−0.371	0.159	0.107	0.000	0.082	0.146	−0.104
DM	0.159	−0.210	0.254	−0.081	−0.268	0.001	−0.217	0.136
VRD flowering	0.319	0.097	−0.205	0.065	−0.128	−0.157	0.047	−0.068
RL0–5	0.029	0.223	0.311	0.039	0.047	−0.049	0.193	0.334
RL5–10	0.093	0.162	0.082	−0.114	−0.039	−0.093	0.551	0.113
RL10–20	0.204	0.266	0.260	0.079	0.104	0.219	0.001	0.070
RL20–40	0.294	0.178	0.148	0.063	0.097	0.253	−0.111	−0.162
RL40–60	0.333	0.103	0.022	−0.051	0.066	0.201	−0.071	−0.242
RL60–75	0.324	0.032	−0.035	−0.155	0.068	0.162	−0.066	−0.189
TRL	0.310	0.204	0.184	−0.004	0.077	0.168	−0.029	−0.055
FRP0–5	−0.128	0.197	0.385	0.091	−0.095	0.006	0.164	0.123
FRP5–10	−0.200	0.134	0.284	0.192	−0.130	−0.059	0.087	−0.173
FRP10–20	−0.213	0.159	0.316	0.199	−0.006	−0.063	−0.190	−0.140
FRP20–40	−0.174	0.100	0.163	0.287	−0.021	−0.173	−0.228	−0.351
FRP40–60	0.152	0.089	−0.140	0.361	−0.020	−0.164	−0.004	0.122
FRP60–75	0.304	0.077	−0.153	0.148	−0.179	−0.254	−0.021	0.041
TFRP	0.255	0.147	−0.089	0.332	−0.178	−0.304	−0.037	0.033

*SCMR, SPAD chlorophyll meter readings; LAI, Leaf area index (m^2^ m^−2^); CB, Canopy biomass (kg ha^−1^); PHI, Pod harvest index (%); PPI, Pod partitioning index (%); HI, Harvest index (%); SW, 100 seed weight (g); GY, Grain yield (kg ha^−1^); Shoot TNC, Shoot TNC (mg g^−1^); Seed TNC, Seed TNC (mg g^−1^); PNA, Pod number per m^2^; SNA, Seed number per m^2^; DM, Days to maturity; VRD Flowering, Visual rooting depth at flowering (cm plant^−1^); RL0–5, Root length at soil depth 0–5 cm (m plant^−1^); RL5–10, Root length at soil depth 5–10 cm (m plant^−1^); RL10–20, Root length at soil depth 10–20 cm (m plant^−1^); RL20–40, Root length at soil depth 20–40 cm (m plant^−1^); RL40–60, Root length at soil depth 40–60 cm (m plant^−1^); RL60–75, Root length at soil depth 60–75 cm (m plant^−1^); TRL, Total root length (m plant^−1^); FRP0–5, Fine root proportion at soil depth 0–5 cm (%); FRP5–10, Fine root proportion at soil depth 5–10 cm (%); FRP10–20, Fine root proportion at soil depth 10–20 cm (%); FRP20–40, Fine root proportion at soil depth 20–40 cm (%); FRP40–60, Fine root proportion at soil depth 40–60 cm (%); FRP60–75, Fine root proportion at soil depth 60–75 cm (%); TFRP, Total fine root proportion (%)*.

**Table 4:** **Eigen values and percent of total variation and component matrix for the principal component axes—Drought stress**.

**Principal components**	**1**	**2**	**3**	**4**	**5**	**6**	**7**	**8**
Eigen values	6.22	3.83	2.74	2.49	1.99	1.28	1.15	1.02
Proportion of variance	0.22	0.14	0.10	0.09	0.07	0.05	0.04	0.04
Cumulative	0.22	0.36	0.46	0.55	0.62	0.66	0.70	0.74
**COMPONENT MATRIX**								
SCMR	0.012	−0.061	0.201	0.052	−0.058	−0.461	−0.361	0.453
LAI	0.157	−0.161	−0.145	−0.024	0.252	0.354	−0.131	0.012
CB	0.131	−0.274	−0.338	0.107	0.021	0.076	−0.240	0.254
PHI	−0.015	−0.157	0.001	0.482	0.094	−0.083	0.020	−0.100
PPI	0.044	0.065	−0.244	0.040	−0.070	0.059	0.362	0.444
HI	−0.059	0.014	0.229	0.295	0.094	−0.313	0.238	−0.413
SW	0.029	0.159	−0.408	−0.102	−0.109	−0.193	−0.063	−0.301
GY	0.114	−0.147	−0.271	0.345	0.016	−0.197	−0.265	−0.127
Shoot TNC	−0.068	−0.038	−0.267	0.180	−0.075	−0.089	−0.005	0.123
Seed TNC	0.016	−0.098	−0.150	0.270	−0.215	0.015	0.474	−0.038
PNA	0.045	−0.331	0.179	0.107	0.071	0.059	0.298	0.192
SNA	0.055	−0.379	0.189	0.288	0.057	−0.004	−0.005	0.117
DM	0.027	−0.167	0.365	−0.142	0.184	0.238	0.075	0.022
VRD Flowering	0.334	0.026	0.083	0.006	−0.251	−0.094	−0.027	−0.016
RL0–5	−0.107	0.264	0.069	−0.021	0.062	−0.253	0.170	0.268
RL5–10	0.169	0.272	−0.103	0.091	0.221	−0.058	0.152	0.218
RL10–20	0.251	0.196	−0.132	0.067	0.302	0.038	0.158	0.024
RL20–40	0.298	0.149	−0.035	0.072	0.236	0.035	−0.018	−0.013
RL40–60	0.330	0.042	0.168	0.028	0.130	−0.051	−0.034	−0.129
RL60–75	0.312	0.018	0.136	0.008	0.104	−0.053	−0.188	−0.074
TRL	0.310	0.228	0.034	0.050	0.264	−0.089	0.068	0.052
FRP0–5	−0.201	0.273	0.187	0.127	0.020	−0.084	−0.098	0.168
FRP5–10	−0.197	0.253	0.088	0.273	0.038	0.148	−0.210	0.050
FRP10–20	−0.195	0.217	0.053	0.311	0.084	0.285	−0.133	−0.009
FRP20–40	−0.170	0.193	−0.026	0.261	0.003	0.335	−0.081	0.003
FRP40–60	0.191	0.108	0.129	0.115	−0.415	0.004	0.080	−0.006
FRP60–75	0.290	0.059	0.077	0.025	−0.312	0.204	−0.073	0.032
TFRP	0.234	0.173	0.141	0.156	−0.406	0.215	−0.064	0.038

*SCMR, SPAD chlorophyll meter readings; LAI, Leaf area index (m^2^ m^−2^); CB, Canopy biomass (kg ha^−1^); PHI, Pod harvest index (%); PPI, Pod partitioning index (%); HI, Harvest index (%); S100W, 100 seed weight (g); GY, Grain yield (kg ha^−1^); Shoot TNC, Shoot TNC (mg g^−1^); Seed TNC, Seed TNC (mg g^−1^); PNA, Pod number per m^2^; SNA, Seed number per m^2^; DM, Days to maturity; VRD Flowering, Visual rooting depth at flowering (cm plant^−1^); RL0–5, Root length at soil depth 0–5 cm (m plant^−1^); RL5–10, Root length at soil depth 5–10 cm (m plant^−1^); RL10–20, Root length at soil depth 10–20 cm (m plant^−1^); RL20–40, Root length at soil depth 20–40 cm (m plant^−1^); RL40–60, Root length at soil depth 40–60 cm (m plant^−1^); RL60–75, Root length at soil depth 60–75 cm (m plant^−1^); TRL, Total root length (m plant^−1^); FRP0–5, Fine root proportion at soil depth 0–5 cm (%); FRP5–10, Fine root proportion at soil depth 5–10 cm (%); FRP10–20, Fine root proportion at soil depth 10–20 cm (%); FRP20–40, Fine root proportion at soil depth 20–40 cm (%); FRP40–60, Fine root proportion at soil depth 40–60 cm (%); FRP60–75, Fine root proportion at soil depth 60–75 cm (%); TFRP, Total fine root proportion (%)*.

## Discussion

This study permitted evaluating shoot and root traits related with drought resistance in a set of 111 RILs of common bean developed for improving drought resistance. Since the study was conducted over three seasons with intermittent drought stress (occurring on and off but especially around the vegetative to early reproductive period of plant development) it facilitated identification of few RILs with superior shoot traits that contributed to improved drought resistance. We complemented the field studies with a greenhouse study on root traits so that we can evaluate the role of root traits in combination with shoot traits for improving drought resistance. Previous research showed that bean genotypes derived from Durango race such as SEA 5 and SEA 15, have mechanisms that can maintain a competitive level of water balance, allowing these genotypes to promote grain formation and filling during drought stress (Rosales et al., 2012; Beebe et al., 2013). By using a set of RILs we could dissect the physiological basis of the superior performance of lines improved for drought resistance. We found significant transgressive segregation for GY and several morpho-physiological shoot and root traits under both irrigated and drought stress conditions. The population distributions were continuous indicating quantitative inheritance for the traits measured.

### Grain yield and canopy biomass

Several shoot traits evaluated in this study showed transgressive segregation in both directions under drought stress, such as GY, CB, SNA, and PHI. Production of CB can be an indicator of the success of the plant in its net fixation of CO_2_, assimilation of nutrients and effective use of water under both optimal and stress conditions, where a higher accumulation of assimilates is reflected in higher rate of crop growth (Bingham, 2001; Araus et al., 2002; Polania et al., 2016a). In various crops, especially in cereals, it has been argued that the potential to increase in the HI may be limited, and therefore future genetic gains in yield potential may depend on an increase in CB production (Bingham, 2001). The identification of genotypes with superior plant growth under both optimal and stress conditions, and the identification of traits to help to a better growth and use of resources would be important to increase genetic gains in breeding programs. In common bean, previous research showed that increase in CB production contributes to increase in grain yield under drought stress (Rosales-Serna et al., 2004; Muñoz-Perea et al., 2007; Klaedtke et al., 2012; Assefa et al., 2013; Beebe et al., 2013; Rao et al., 2013, 2016a; Polania et al., 2016a,c). It has also been reported that CB accumulation over time is sensitive to drought stress, as result of reduced transpiration and net photosynthesis (Klaedtke et al., 2012; Mir et al., 2012; Rosales et al., 2012; Rao et al., 2013; Polania et al., 2016a). In this study CB production was reduced by 36% under intermittent drought stress compared with irrigated conditions. Our results confirmed previous research that improved CB production contributes to better GY under both irrigated and drought stress conditions, based on the positive and highly significant correlation between GY and CB (Table 1).

Several lines were outstanding in CB production and GY under drought stress and some of these lines also combined deep rooting ability with GY under drought stress. These lines were able to access more water, with the help of their root system, combined with increased photosynthate mobilization (HI and PHI), resulting in better resistance to drought (Polania et al., 2012, 2016a,c; Assefa et al., 2013; Rao et al., 2013, 2016a; Beebe et al., 2014; Rao, 2014). A few RILs presented higher values of CB under drought stress, combined with moderate values of GY indicating that a high value of CB alone is not enough to have higher GY under drought stress. Photosynthate remobilization ability for pod and grain formation was lower in these lines.

Some RILs were superior in their GY under drought stress but they did not produce adequate CB compared with the other genotypes tested. This indicates the importance of the efficiency of mobilization of photosynthates from vegetative plant structures to pod production in these lines (Figures 2, 3). In common bean, it seems that combination of plant attributes such as deep rooting ability, rapid plant growth rate, and an efficient resource management by the plant, will permit greater biomass accumulation under both irrigated and drought stress, and result in higher GY under both irrigated and drought conditions. Adequate CB accumulation under both optimal and drought stress conditions is important to ensure availability and supply of photoassimilates to pod and seed formation.

### Grain yield, photosynthate remobilization, and sink strength

Pod partitioning index (PPI) has been reported as an useful index to determinate the remobilization from vegetative structures to pod production in common bean (Klaedtke et al., 2012; Assefa et al., 2013; Beebe et al., 2013, 2014; Rao et al., 2013, 2016a; Polania et al., 2016a). PPI can be overestimated because it was based on the CB-values at mid-pod filling growth stage with the assumption that this growth stage reflects the maximum vigor. The values of CB may be underestimated particularly under irrigated and intermittent drought conditions, because of possible additional vegetative growth occurring after mid-pod filling to physiological maturity due to irrigation or rainfall. The distribution of rainfall in the 3 years of evaluation indicate that the crop suffered intermittent drought stress, some years the rainfall during grain filling stage was higher than the other years (Figure 1). This additional water availability can cause additional vegetative growth that is difficult to estimate (due to leaf fall during this period). We assume that the plant can take advantage of this additional water to improve grain formation and filling which could result in better grain filling under intermittent than terminal drought stress. The correlation coefficients in this study between GY and PPI were not positive and significant, possibly due to the effect of additional rainfall, especially 2007 compared to the other 2 years. These conditions may make some lines to revert back to the behavior of wild bean (Beebe et al., 2008) exhibiting different patterns of growth and remobilization, making it unclear the major contribution of this trait. However, several RILs combined superior GY with PPI under intermittent drought stress indicating their superior ability to remobilize photosynthates from vegetative plant structures to pod production.

The contribution of remobilization of photoassimilates from vegetative structures to the pod and grain production for improving drought resistance has been reported either by estimating dry matter partitioning indices such as HI, PPI, and PHI (Hall, 2004; Rosales-Serna et al., 2004; Klaedtke et al., 2012; Rosales et al., 2012; Assefa et al., 2013; Rao et al., 2013, 2016a; Beebe et al., 2014; Rao, 2014; Polania et al., 2016a,c) or by quantifying starch and sugar accumulation and partitioning (Cuellar-Ortiz et al., 2008; Rosales et al., 2012; Andrade et al., 2016). Field evaluation in this study over three seasons under intermittent drought stress showed stronger correlation between PHI and GY confirming the contribution of mobilization of photosynthates from pod walls to grain (Table 1). It is important to point out that while several lines were superior in their PHI and GY, two RILs (MR 12 and MR 112) were high yielding under drought stress but presented thicker pod walls (relatively lower than average values of PHI; Figure 4). These two lines had relatively higher values of CB and SNA that contributed to greater GY under drought stress. Improving the values of PPI and PHI in these two lines could improve further GY-values of these lines under drought stress. These two lines could further improve their GY-values under drought stress by enhancing their remobilization ability of photosynthates from pod wall to seed filling (i.e., increase in PHI). These results are consistent with previous reports which suggested that PHI could serve as a useful selection criteria for improving drought resistance in common bean because of its simplicity in measurement, significant correlation with GY under both irrigated and drought conditions and high heritability (Assefa et al., 2013; Rao et al., 2013; Beebe et al., 2014; Polania et al., 2016a).

Drought stress is known to reduce yield components such as PNA and SNA (Rao et al., 2013; Assefa et al., 2015). Seed number per pod has been identified as useful criteria for selection for improving drought resistance because of its higher heritability and contribution to genetic gain (Ramirez-Vallejo and Kelly, 1998). The decrease in the formation of pods and grains under drought stress is due to several factors, including pollen grain sterility that reduces pollen grain germination and pollen tube growth, and inadequate photosynthate supply that prevents embryo development (Farooq et al., 2016). Selection of genotypes that have greater sink strength reflected in greater values of SNA is required to increase GY under drought conditions. Our results demonstrate this relationship to be a positive and significant correlation between SNA and GY under drought stress. However, it is noteworthy that there are genotypes that have higher values of SNA but lower values of GY under drought stress (Figure 5), indicating that these genotypes are failing in their ability to optimize photosynthate mobilization to support grain filling process. This behavior can be evidenced in the relationship between SNA and PHI (Figure 6), in which genotypes such as MD 23–24, MR 114, MR 12, MR 11, and MR 35 showed higher SNA but lower PHI under drought conditions. Thus, these genotypes are capable of setting seeds but are poor in their ability to fill the seeds. An increase in photosynthate remobilization together with improved sink strength was observed in the superior genotypes that combined higher values of GY with SNA and PHI under drought stress conditions (Figures 5, 6).

### Grain yield and physiological efficiency through early maturity

The significant negative relationship between GY and DM under drought stress (Table 1) indicated that early maturing genotypes were more adapted to drought stress. The common bean farmers have multiple reasons for preferring short season varieties, an important one among them is to minimize exposure to drought (White and Singh, 1991; Beebe, 2012). Earliness is more useful where terminal drought predominates (Beebe et al., 2014) but a shorter growth cycle can reduce GY potential per day by an estimated value of 74 kg ha^−1^ (White and Singh, 1991). This penalty in GY per day could be markedly reduced or even completely eliminated through improved physiological efficiency of the plant through genetically improving the capacity of the plant to produce more seeds and especially together with the ability to have better filling of these seed under drought stress (i.e., greater sink strength). In common bean different field studies showed that early maturing genotypes with superior photosynthate remobilization ability can yield better under both drought and irrigated conditions (Klaedtke et al., 2012; Rao et al., 2013, 2016a; Beebe et al., 2014; Polania et al., 2016a,c). This improved physiological efficiency under drought stress was shown to be independent of yield potential and phenological plasticity (Polania et al., 2016c). The phenotypic correlations between GY and CB, PHI, DPM, SNA, and SW suggest that in common bean higher values of CB combined with an efficient remobilization of photosynthates to the pod and grain formation could contribute to greater sink strength through higher values of both SNA and SW.

### Grain yield and root traits

Root growth and shoot growth have a complex relationship. In general, the shoot provides the root with carbon and certain hormones, and the root provides the shoot with water, nutrients, and also with hormones. To increase grain yield through a better plant growth under both optimal and drought stress conditions, the root system must be able to supply water and nutrients to the new plant growth without sequestering too much photoassimilates from the shoot (Bingham, 2001). Defining the morpho-physiological traits and mechanisms that are suited to different agroecological niches will play an important role in the development of new varieties adapted to different types of drought stress. Different studies on common bean and other crops contributed to define the root system characteristics for superior resistance to drought (Lynch, 2013). Among the root characteristics evaluated in this study, none stood out for its outstanding contribution or correlation with more grain production under drought stress and even under irrigated conditions (Table 1). This indicates the complexity of the relationship between root system development and drought resistance.

Results from this study demonstrated transgressive segregation in both directions under drought stress in several root traits evaluated, such as total root length production, deep root production, visual rooting depth, and fine root proportion. Several RILs exhibited markedly superior or lower expression of root traits than both parents (SEA 5 and MD 23–24) under drought as well as irrigated conditions. Transgressive segregation in root traits in common beans under irrigated and drought conditions have been observed also in RILs population of BAT 477 × DOR 364 (Asfaw and Blair, 2012). It was observed in several drought resistant RILs that root production is stimulated by drought stress compared with irrigated conditions and this could be an adaptive response of the plant to drought stress (Turner, 1979; Rao, 2014).

Results on phenotypic correlations and the multivariate analysis using the data on different shoot and root traits indicated that improved resistance to intermittent drought stress in common bean could result from different plant strategies. These strategies include different combinations of shoot and root traits that allows the plant to better adapt to drought stress. Based on the phenotypic differences in GY, CB, VRD and TRL, we classified the drought resistant lines into two groups, water savers and water spenders (Table 5). The water spender type genotypes combined higher values of GY with SNA under drought stress with rapid development of deep root system that allows the plant a faster access to available water in deep soil profiles (Table 5). This response to drought allows to continue the processes of gas exchange and carbon accumulation and facilitates improved remobilization of photosynthates resulting in an increased values of SNA and GY under drought stress. This overall response at whole plant level reflects an EUW rather than improved WUE resulting from partial closure of stomata (Polania et al., 2016a). Results from water spender type genotypes indicate that a deeper and vigorous root system helps to the plant to support a better sink strength that was reflected in higher values of SNA under drought conditions (Table 5). These water spender type genotypes would be better suited to intermittent drought conditions where water could be available at depth.

**Table 5:** **Combination of shoot and root traits of bean lines classified such as water saver type, water spender type and drought susceptible type that were grown under drought stress under greenhouse and field conditions at CIAT–Palmira, Colombia**.

**Genotype**	**Grain yield (kg ha**^**−1**^**)**		**Canopy biomass (kg ha**^**−1**^**)**		**Pod harvest index (%)**		**Pod partitioning index (%)**		**Seed number per area (m**^**2**^**)**		**Visual rooting depth at flowering (cm plant**^**−1**^**)**		**Root length at soil depth 60–75 cm (m plant**^**−1**^**)**		**Total root length (m plant**^**−1**^**)**	
	**Irrigated**	**Drought**	**Irrigated**	**Drought**	**Irrigated**	**Drought**	**Irrigated**	**Drought**	**Irrigated**	**Drought**	**Irrigated**	**Drought**	**Irrigated**	**Drought**	**Irrigated**	**Drought**
**DROUGHT RESISTANT LINES WITH DEEP ROOTING ABILITY (WATER SPENDER TYPE)**																
MR 81	1,954	1,546	4,433	2,771	83	81	54	47	1,277	1,115	69	63	2.3	2.7	52	41
MR 25	2,117	1,523	4,665	3,121	79	76	71	53	1,187	1,058	75	75	5.9	4.8	55	54
MR 95	1,943	1,471	4,865	2,850	79	79	45	53	1,384	1,029	69	70	4.1	3.2	50	42
MR 93	2,088	1,462	4,213	3,172	79	78	57	57	1,217	1,099	72	75	4.8	5.5	52	50
MR 67	2,001	1,363	4,464	2,788	79	77	59	69	1,286	838	68	75	6.7	5.7	39	58
SEA 5	1,763	1,284	4,787	3,124	77	75	66	54	1,042	782	75	73	2.6	5.2	37	43
**DROUGHT RESISTANT LINES WITHOUT DEEP ROOTING ABILITY (WATER SAVER TYPE)**																
MR 112	1,996	1,539	4,302	2,982	77	74	78	59	1,271	996	58	58	0.3	0.4	35	21
MR 24	1,881	1,453	4,864	3,034	80	77	67	55	1,124	973	56	55	2.1	0.4	47	38
MR 77	2,018	1,412	4,509	3,259	80	78	67	73	1,256	1,086	69	66	2.0	0.7	34	35
MR 120	2,123	1,405	5,086	3,186	82	80	61	57	1,006	741	60	57	0.4	0.0	37	30
MR 75	1,977	1,403	4,395	2,604	80	77	66	62	1,143	793	64	63	0.6	1.2	44	39
MR 110	1,980	1,391	4,653	3,115	81	79	57	73	1,123	1,017	71	62	2.8	0.6	54	32
**DROUGHT SUSCEPTIBLE TYPE GENOTYPES**																
MR 69	1,945	987	4,021	2,427	81	78	55	58	1,297	1,039	56	54	0.8	0.3	33	27
MR 54	1,604	947	3,710	2,707	79	74	70	71	1,133	822	55	55	1.7	0.7	34	27
DOR 390	2,075	900	4,593	2,443	81	75	70	62	1,432	970	68	54	2.6	0.7	33	24
MR 116	1,755	846	3,500	2,494	80	71	63	53	1,332	763	66	70	0.7	0.5	49	33
MR 109	1,368	833	3,478	2,963	79	74	46	58	1,017	713	74	60	2.9	2.8	44	38
EAP 9,510-77	1,957	708	4,558	2,177	80	66	51	51	1,259	663	48	65	0.1	2.3	27	38

The strategy of water spender type genotypes under intermittent drought stress conditions may be that the deeper roots with better water extraction capacity can support the rate of photosynthesis and the accumulation of water soluble carbohydrates in the stem and this accumulated photosynthate could be remobilized to grain filling (Lopes and Reynolds, 2010). Several studies have demonstrated the contribution of deep rooting in increased water extraction from lower soil depth and its relationship with superior resistance to drought (Sponchiado et al., 1989; White and Castillo, 1992; Lynch and Ho, 2005; Ryser, 2006; Polania et al., 2009, 2012; Asfaw and Blair, 2012; Beebe et al., 2013; Rao, 2014; Rao et al., 2016b). Some efforts have been made in identifying genes and molecular markers that are associated with deep rooting ability (Asfaw and Blair, 2012) where a RILs population of DOR 364 × BAT 477 was used to identify quantitative trait loci (QTLs) that were associated with root traits under drought stress. Several QTLs were identified on linkage groups b01 or b11, which explained up to 41% of genetic variance.

The water saver type genotypes were superior in GY with moderate values of SNA under drought stress but their root system was slower in its development (Table 5). It is possible that these genotypes were better adapted to drought because of their ability to regulate stomatal opening for improved transpiration efficiency while maintaining their capacity to remobilize photosynthates toward pod and grain production (Polania et al., 2016a). These water saver type genotypes, would be suitable to semi-arid to arid environments where water availability is very limited with longer terminal drought stress conditions. Thus, further research on water use, photosynthesis and carbon mobilization is needed on the entire RIL population to classify the RILs as water savers or as water spenders and also to identify QTLs for different water use patterns based on both shoot and root traits.

Shallower rooting ability under drought stress can be complemented with traits related with conserving water at vegetative stage, such as lower leaf conductance, smaller leaf canopy, that would make more water available for reproductive growth and grain filling, resulting in better GY under terminal drought stress conditions (Zaman-Allah et al., 2011; Araújo et al., 2015). A poor root system can be limiting for an optimal plant development and grain production under drought stress, even for a good response in optimal conditions; as can be evidenced in the drought sensitive genotypes (Table 5). These genotypes showed less resistance to drought (lower values of GY) and were characterized by lower values of total root production as well as less proportion of roots at deeper soil layers under both irrigated and drought conditions. However, some RILs had deep root system but not higher values of GY thus indicating that deep rooting alone with lower sink strength will not result in higher values of GY under drought.

The results from both the field and greenhouse studies indicated the need to determine what size and what kind of distribution of root system is required in a certain field site to avoid a trade-off or any restriction in the plant growth and yield (Bingham, 2001). A very vigorous root system, in an inefficient plant to assimilate CO_2_, becomes plant's another sink, competing for photoassimilates with the economic organ of interest of the plant, and increase the sensitivity to drought stress. Thus, a vigorous and deeper root system, with rapid growth is useful but not enough to have greater resistance to drought in common bean. It is the strategic combination of traits that improves physiological efficiency such as a better developed root system helping the plant to access water to maintain transpiration rates and vegetative growth combined with the ability to remobilize photosynthates from vegetative structures to the pods and subsequently to grain production is what is needed for improved drought resistance (Beebe et al., 2014; Rao, 2014; Araújo et al., 2015; Polania et al., 2016a; Rao et al., 2016a).

## Conclusions

We evaluated different root and shoot traits in a large RILs population and identified a few relevant traits for improved resistance to intermittent drought in common bean. The phenotypic data generated from this work will be useful to identify shoot and root QTLs associated with improved resistance to intermittent drought. Previous studies have reported the contribution of individual traits such as deep rooting, CB, water use, HI and PHI to the adaptation to drought stress in common bean, but not the combination of these traits and how the combination particularly with a focus on sink strength contributes to improved adaptation to intermittent drought stress. Our results indicate that common bean genotypes respond to drought stress as water spending types or water saver types. The water saver type of genotypes respond to drought with intermediate to shallow rooting system, high water use efficiency, reduced sink strength, and superior photosynthate remobilization to pod and grain formation. The water spender type of genotypes respond to drought with a better developed root system helping the plant to access water to maintain transpiration rates and vegetative growth, combined with the ability to remobilize photosynthates from vegetative structures to the pods and subsequently to seed production resulting in a superior number of pods and seeds per area. We observed transgressive segregation in root traits such as total root length and deep rooting ability under irrigated and drought conditions. We identified five RILs (MR 25, MR 93, MR 67, MR 81, MR 95) as drought resistant-water spender types and five RILs (MR 112, MR 24, MR 77, MR 120, MR 75) as drought resistant-water saver types. We identified rooting depth, CB, PPI, PHI, PNA, and SNA as useful traits for improving resistance to intermittent drought. Some of these traits are easier to implement in a breeding program due to their simplicity and relatively low analytical cost such as PHI.

## Author contributions

JP, CC, SB, and IR designed the experiments and contributed to data interpretation. JP, CC, MG, MR, and FV collected and analyzed the data. All authors read and approved the final manuscript.

### Conflict of interest statement

The authors declare that the research was conducted in the absence of any commercial or financial relationships that could be construed as a potential conflict of interest.
